# Functional changes in long-term incubated rat precision-cut lung slices

**DOI:** 10.1186/s12931-022-02169-5

**Published:** 2022-09-20

**Authors:** Sarah Marie Nußbaum, Julia Krabbe, Svenja Böll, Aaron Babendreyer, Christian Martin

**Affiliations:** 1 Institute of Pharmacology and Toxicology, Medical Faculty, RWTH Aachen University, Wendlingweg 2, 52074 Aachen, Germany; 2 Institute of Occupational, Social and Environmental Medicine, Medical Faculty, RWTH Aachen University, Pauwelsstraße 30, 52074 Aachen, Germany; 3 Department of Pediatrics, Medical Faculty, RWTH Aachen University, University Hospital Aachen, Pauwelsstraße 30, 52074 Aachen, Germany; 4 Institute of Molecular Pharmacology, Medical Faculty, RWTH Aachen University, Wendlingweg 2, 52074 Aachen, Germany

**Keywords:** Precision-cut lung slices, PCLS, Rat, Long-term incubation, Viability, Histopathology, Bronchoconstriction, Gene expression, Fetal bovine serum, FBS

## Abstract

**Background:**

Respiratory diseases represent a global health burden. Because research on therapeutic strategies of airway diseases is essential, the technique of precision-cut lung slices (PCLS) has been developed and widely studied. PCLS are an alternative ex vivo model and have the potential to replace and reduce in vivo animal models. So far, the majority of studies was conducted with short-term cultivated PCLS (≤ 72 h). As there is large interest in research of chronic diseases and chronic toxicity, feasibility of cultivating human PCLS long-term over 2 weeks and recently over 4 weeks was investigated by another research group with successful results. Our aim was to establish a model of long-term cultivated rat PCLS over a period of 29 days.

**Methods:**

Rat PCLS were cultured for 29 days and analysed regarding viability, histopathology, reactivity and gene expression at different time points during cultivation.

**Results:**

Cultivation of rat PCLS over a 29-day time period was successful with sustained viability. Furthermore, the ability of bronchoconstriction was maintained between 13 and 25 days, depending on the mediator. However, reduced relaxation, altered sensitivity and increased respiratory tone were observed. Regarding transcription, alteration in gene expression pattern of the investigated target genes was ascertained during long-term cultivation with mixed results. Furthermore, the preparation of PCLS seems to influence messenger ribonucleic acid (mRNA) expression of most target genes. Moreover, the addition of fetal bovine serum (FBS) to the culture medium did not improve viability of PCLS. In contrast to medium without FBS, FBS seems to affect measurements and resulted in marked cellular changes of metaplastic and/or regenerative origin.

**Conclusions:**

Overall, a model of long-term cultivated rat PCLS which stays viable for 29 days and reactive for at least 13 days could be established. Before long-term cultivated PCLS can be used for in-depth study of chronic diseases and chronic toxicity, further investigations have to be made.

**Supplementary Information:**

The online version contains supplementary material available at 10.1186/s12931-022-02169-5.

## Background

Respiratory diseases such as chronic obstructive pulmonary disease (COPD) or acute respiratory infections are among the leading causes of death worldwide [1] and represent a global health burden. According to estimates from 2019 262 million people suffered from asthma, while prevalence of COPD amounted to 212 million worldwide [2]. Both chronic respiratory diseases cause high economic and social costs due to disability and premature mortality. This includes direct costs regarding drugs and health service as well as indirect costs related to lost production [3]. All these facts make further research on therapeutic strategies of those chronic respiratory diseases essential.

In recent years, there has been a large interest in organotypic tissue models. Therefore, the technique of precision-cut lung slices (PCLS) was thoroughly investigated in respiratory research. PCLS are viable 3D lung tissue slices of approximately 250 µm thickness with intact microanatomy and preserved cellular composition [4, 5]. Besides investigations to airway functionality [4, 6–8], measurements of vascular effects [9–11] and mucociliary function [12, 13], PCLS are used for pharmacological and toxicological studies [14–16]. Furthermore, PCLS can be used to evaluate effects of sensitisation [13, 17], asthma [18], COPD [19], fibrosis [19] and infectious diseases [20, 21]. Especially human PCLS of relatively healthy and diseased patients represent a valuable tool to determine differences in functional studies and possible pharmacological treatments [22].

The majority of studies was conducted with PCLS cultured for a short period of time (≤ 72 h) [7, 13, 23] and was thus limited to issues regarding acute effects of drugs and chemicals. Aside from these short-term experiments there is large interest in research of drugs’ chronic effects as well as investigations of mechanisms and methods of treatment regarding chronic airway diseases (e.g., asthma, COPD). For this reason another research group tested feasibility of long-term cultivated human PCLS as a chronic model over a period of 2 weeks [24, 25]. They reported sustained viability, reactivity and structural integrity of PCLS for the investigated time period. Recently, viability of human PCLS for up to 4 weeks could be shown, however, a continuous cell loss, decrease in ciliary beat and increasing manifestation of keratinizing squamous metaplasia over time were observed [26].

Aim of the current study was to establish a model of long-term cultivated rat PCLS over a period of 29 days. Regarding long-term cultivation several questions arose which we will address in the manuscript:

First, do PCLS stay viable and free of contamination over a period of 29 days and if so, do the slices stay reactive and maintain their ability of bronchoconstriction? Second, are there any changes in gene expression pattern and viability which could explain possible effects on bronchoconstriction and can viability be influenced by the addition of fetal bovine serum (FBS)? Finally, does the preparation of PCLS effect basal gene expression?

## Materials and methods

### Laboratory animals

Lungs were taken from female Wistar rats (305 ± 20 g) obtained from Janvier Labs (Le Genest-Saint-Isle, France). The animals were kept under controlled conditions (22 °C, 55% humidity, 12 h day/night rhythm) and received laboratory food and tap water ad libitum.

### Culture media and reagents

Low-melting agarose was obtained from Sigma-Aldrich Chemie GmbH (Steinheim, Germany). The culture medium consisted of minimal essential medium (pH 7.2) composed of CaCl_2_, MgSO_4_, KCl, NaCl, NaH_2_PO_4_, glucose, NaHCO_3_ (all from Sigma-Aldrich), HEPES (Carl Roth GmbH + Co. KG, Karlsruhe, Germany), sodium pyruvate, glutamine (both from Capricorn Scientific GmbH, Ebsdorfergrund, Germany), amino acids and vitamins (both from Sigma-Aldrich) and was supplemented with penicillin and streptomycin (Sigma-Aldrich) (Additional file 1: Table S1) as described before [27]. The slicing medium consisted of the same components except for sodium pyruvate, glutamine, amino acids, vitamins, penicillin and streptomycin. Besides, a culture medium with the addition of 10% heat-inactivated and sterile-filtered FBS (Thermo Fisher Scientific, Life Technologies Europe BV, Bleiswijk, Netherlands, Gibco 10,270) was used for viability tests.

### Preparation of PCLS

PCLS were prepared as described before [28] with some modifications. Briefly, intraperitoneal anaesthesia was performed with 60 mg/kg Pentobarbital-Sodium (Narcoren; Merial GmbH, Hallbergmoos, Germany) and verified by missing reflexes. Afterwards, the abdomen was opened and the rat exsanguinated. Thereafter, thoracotomy and tracheotomy followed. *Lobus accessorius* of the right lung was tied off to prevent the lobe from filling with agarose. The lung was inflated with 15–20 ml of warm 1.5% low-melting agarose via the trachea depending on the weight of the animal and until a slight resistance developed and later solidified on ice. After polymerisation of the agarose, the lung was removed *en bloc*, the lobes were separated and tissue cores with a centred airway perpendicular to the long axis were prepared with a rotating sharpened metal tube (diameter: 10 mm). Approximately 250–300 µm thin slices were cut with a microtome (Krumdieck Tissue Slicer, Alabama Research and Development, Munford, AL, USA). Slices were placed into Petri dishes with warmed culture medium and incubated at 37 °C in a humid 5% carbon dioxide atmosphere. After preparation, the medium was changed several times within the first 4 h to remove cell debris as well as released mediators from the tissue. On the next day, the slices were placed into 24-well tissue culture plates in 1 ml of culture medium. The plates were maintained under tissue culture conditions and the medium was changed every day. Contamination status of the PCLS culture was controlled daily by visual inspection and monitoring the supernatant by culture-based assays. For measurements, only slices with an airway free of agarose, beating cilia and intact and relaxed airway smooth muscle (ASM) layer were used.

### Viability tests

To assess viability of long-term cultivated PCLS, water soluble tetrazolium (WST)-1 and lactate dehydrogenase (LDH) assays were performed with tissue slices incubated in a standard culture medium (named PCLS in the absence of serum) and others incubated in a culture medium with the addition of 10% FBS (named PCLS in the presence of serum) as the effects of FBS on long-term cultivated slices were of interest as well. Starting on day 1 after preparation, viability tests were carried out every 4^th^ day over a period of 29-day long-term cultivation.

#### WST-1 assay

The metabolic activity of PCLS was determined using the WST-1 reagent from Roche Diagnostics GmbH (Mannheim, Germany). The medium was removed and replaced with 250 µl of a freshly prepared WST-1 solution (1:10 dilution in culture medium). After incubation for 1 h at 37 °C, 100 µl of supernatant were transferred to a 96-microtiterplate in duplicates. Absorbance was determined at 450 nm with a reference wavelength of 595 nm.

#### LDH assay

LDH activity in PCLS was determined in culture supernatants using the Cytotoxicity Detection Kit (LDH) obtained from Roche. Besides LDH release (LDHr) over 24 h, the maximal LDH activity (= total LDH, LDHt) was of interest as well. Therefore, 200 µl medium of half of the slices in both groups (PCLS in the absence and presence of serum, respectively) were removed and replaced with 200 µl 1% Triton (Merck KGaA, Darmstadt, Germany). After incubation for 30 min at 37 °C, 50 µl of supernatant of all slices were transferred to a 96-microtiterplate in duplicates. 50 µl LDH reaction mixture were added and incubated for 20 min at room temperature protected from light. Absorbance was determined at 492 nm with a reference wavelength of 595 nm. For the calculation of relative cytotoxicity, the ratio of LDHr/LDHt was determined.

### Histopathologic analysis

After cultivation, PCLS were fixed in buffered formalin, embedded in paraffin and sliced into 4–5 µm slices. The paraffin slices were stained with hematoxylin and eosin (HE) using standard histological procedures. Magnifications and scale bars are indicated in the figure and description. The histomorphological evaluation was performed semiquantitatively according to the following criteria: The respiratory epithelium of airways was completely adherent at the lamina propria or tunica adventitia (score 0), detached in one airway per PCLS (score 1) or detached in more than one airway per PCLS (score 2). The alveolar septal structure was scored as no cell loss within the alveolar septa (score 0), rare cell loss (score 1), moderate cell loss with many lost alveolar cells (score 2) or severe cell loss when most of the alveolar cells were lost (score 3). In addition, histopathologic abnormalities were assessed according to the frequency of occurrence in the punch area (score 0–3).

### Measurement and imaging of bronchoconstriction

Over a period of 29 days cumulative concentration–response-curves (CRCs) were performed under a hood with acetylcholine (Ach; 10^–8^–10^–3^ M; Sigma-Aldrich Chemie GmbH, Taufkirchen, Germany), endothelin-1 (ET-1; 10^–9^–10^–6^ M; Bachem AG, Bubendorf, Switzerland) and 9,11-dideoxy-9α,11α-methanoepoxy prostaglandin F_2α_ (U46619; 10^–10^–10^–5^ M; Cayman Chemical, Ann Arbor, MI, USA) every 4th day—starting on day 1 after preparation. Images were recorded every 5 s for a time period of 5 min for Ach, every 10 s for a time period of 10 min for ET-1 and U46619 until the next concentration was added. The same slices for all measurements of the specific mediator during long-term cultivation with an average of two slices per long-term experiment was used.

For measurement of bronchoconstriction, PCLS were placed into a culture dish and were fixed by a platinum wire at the bottom of the dish to avoid moving during measurement. Airways were imaged using a self-built microscope (Institute of Pharmacology and Toxicology, Medical Faculty, RWTH Aachen University, Germany) with a USB camera on the top and a diffuse light source below the sample. Image analysis was performed using ImageJ (U.S. National Institutes of Health, USA). The airway area before addition of the lowest concentration of the agonist was defined as 100%. Bronchoconstriction was expressed as the percentage of the initial airway area (IAA). The airway area was analysed based on the IAA of the current measuring day and that of the first measuring day. GraphPad Prism 9 software (GraphPad Software, San Diego, CA, USA) was used for fitting sigmoidal CRCs. Efficacy and half-maximal response (EC_50_) as well as maximal airway area (at each measurement day compared to the first day) were calculated by nonlinear regression with a four-parameter logistic equation. Data is expressed as mean ± standard deviation (SD).

### RNA isolation

To analyse gene expression and possible changes over the period of long-term cultivation, a specific number of slices has been snap frozen in liquid nitrogen on the measuring days of bronchoconstriction. Besides, *Lobus accessorius* has been snap frozen on the day of preparation. Slices and lobe were pulverized in liquid nitrogen with a mortar and pestle, 100 mg of the slices and 20 mg of the lobe, respectively, were used for isolating ribonucleic acid (RNA). Preliminary tests (unpublished data) showed that a combination of phenol–chloroform extraction (RNA Solv Reagent, Omega Bio-tek Inc., Norcross, GA, USA) and magnetic-bead technique (MagMAX-96 for Microarrays Total RNA Isolation, Invitrogen, Thermo Fisher Scientific Baltics UAB, Vilnius, Lithuania) achieved best results regarding RNA yield and quality. Therefore, the whole aqueous phase of phenol–chloroform extraction (based on the protocol of Toni et al. 2018 [29] with a few modifications) was transferred in microreaction tubes and RNA was cleaned up with magnetic beads according to the supplier’s instructions (Spin Procedure) with an additionally integrated deoxyribonuclease (DNase) treatment. RNA yield (concentration) and purity (A_260_/A_280_ and A_260_/A_230_ ratio) were measured by spectrophotometry (NanoDrop 1000 Spectrophotometer, PEQLAB Biotechnologie GmbH, Erlangen, Germany), RNA integrity was analysed by 1% agarose gel electrophoresis (Gel Doc XR + , Bio-Rad Laboratories GmbH, Feldkirchen, Germany).

The isolated RNA from PCLS achieved sufficient yields (concentration: 77.60 ± 27.71 ng/µl) and was of good quality, as evaluated by the A_260_/A_280_ ratio (2.039 ± 0.073). Those from the native lung tissue even obtained a concentration of 414.62 ± 49.16 ng/µl with a similar A_260_/A_280_ ratio (2.070 ± 0.018). Regarding the A_260_/A_230_ ratio the isolated RNA from PCLS obtained a ratio of 1.180 ± 0.277, the native lung tissue a ratio of 1.870 ± 0.317. The RNA of all samples possessed a high degree of integrity with two distinct bands in the gel electrophoresis representing the prominent 28S and 18S ribosomal RNAs.

### RT-qPCR

Reverse transcription (RT) of RNA was performed using Prime Script RT Master Mix (Perfect Real Time) (Takara Bio Europe, Gothenburg, Sweden), according to the manufacturer’s instructions, with an input of 230 ng RNA. For quantitative polymerase chain reaction (qPCR) iTaq Universal SYBR Green Supermix (Bio-Rad) was used according to the supplier’s manual. The specific primers (Kaneka Eurogentec S.A., Seraing, Belgium) and annealing temperatures are listed in the (Additional file 2: Table S2). The qPCR runs were performed by CFX Connect Real-Time PCR Detection System (Bio-Rad) with the following protocol: 40 cycles of 10 s denaturation at 95 °C, 10 s annealing at the indicated temperature and 15 s amplification at 72 °C. Relative quantification was conducted with the CFX Maestro Software 2.0 (Bio-Rad). The messenger ribonucleic acid (mRNA) levels were quantified by RT-qPCR analysis and normalised to the mRNA level of reference genes. The most stable reference genes were determined with the geNorm algorithm of CFX Maestro Software (Reference Gene Selection Tool, Bio-Rad). Based on the results, ribosomal protein lateral stalk subunit P0 (Rplp0) and beta-actin (Actb) were chosen as the most stable reference genes. As normalisation was performed against these two reference genes, the term “Reference gene index (Ref. gene index)” was introduced to make graphs clearer.

### Statistical analysis

Statistics were carried out using the general mixed model analysis (PROC GLIMMIX, SAS 9.4, SAS Institute Inc., Cary, NC, USA). The data were analysed for the optimal distribution using the Akaike Information Criterion, the Bayesian Information Criterion, residual plots and the Shapiro–Wilk test. If necessary, laboratory animals were set as random term and blocking was used to assess for animal-specific differences. In case of heteroscedasticity (based on covtest statement) the degrees of freedom were adapted by the Kenward-Roger approximation. If data still did not show (log)normal distribution (based on Shapiro–Wilk test), a non-parametric Kruskal–Wallis test was performed (GraphPad Prism 9). Multiple comparisons were corrected by false discovery rate, significant p-value was < 0.05.

## Results

### Long-term cultivation of PCLS

Most (~ 93.5%) PCLS were free of contamination for the entire duration of 29 days, with only slices from one animal being contaminated after a few days in culture.

### Viability of PCLS during long-term cultivation

#### WST-1 assay

**Fig. 1:**
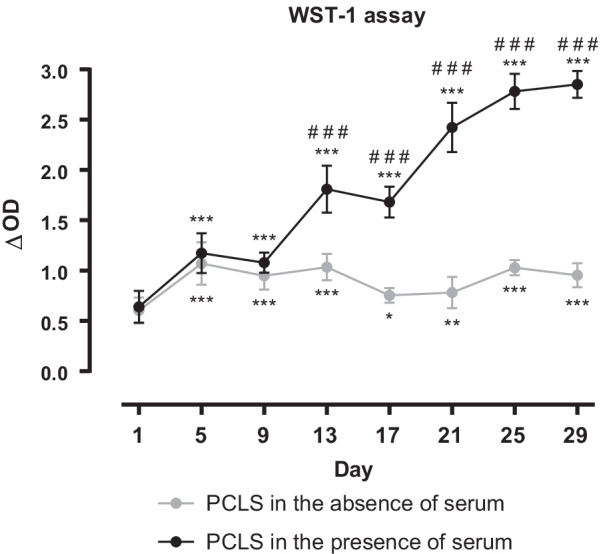
Metabolic activity of long-term cultivated rat precision-cut lung slices (PCLS). Metabolic activity of tissue PCLS incubated in a standard culture medium (named PCLS in the absence of serum) and others incubated in a culture medium with the addition of 10% fetal bovine serum (FBS) (named PCLS in the presence of serum) was measured using water soluble tetrazolium (WST)-1 assay (optical density (OD) 450 nm, reference wavelength 595 nm). For each timepoint, measurements were performed in duplicates, n = 3. Data is expressed as mean ± standard deviation (SD). Statistics were carried out using the general mixed model analysis (PROC GLIMMIX, SAS 9.4, SAS Institute Inc., Cary, NC, USA), statistical differences to day 1 are indicated by asterisks (*0.01 ≤ p < 0.05; **0.001 ≤ p < 0.01, ***p < 0.001), statistical differences between the two groups (PCLS in the absence vs. PCLS in the presence of serum) are indicated by hashes (^###^p < 0.001)

Mitochondrial metabolic activity of PCLS in the absence of serum, measured by WST-1 assay, was lowest on day 1 after preparation (Fig. 1). After an increase until day 5 metabolic activity remained on the same level for the remaining time of long-term cultivation—except for a slight decrease on days 17 and 21.

Metabolic activity of PCLS in the presence of serum was similar in shape to that of PCLS in the absence of serum for the first nine days in culture, although on a slightly higher level. From day 13 onwards, a significant increase in metabolic activity (compared to PCLS in the absence of serum) was observed: On day 17 ΔOptical density (OD) of PCLS in the presence of serum was twice as high, on day 29 even three times higher than that of PCLS in the absence of serum.

#### LDH assay

**Fig. 2:**
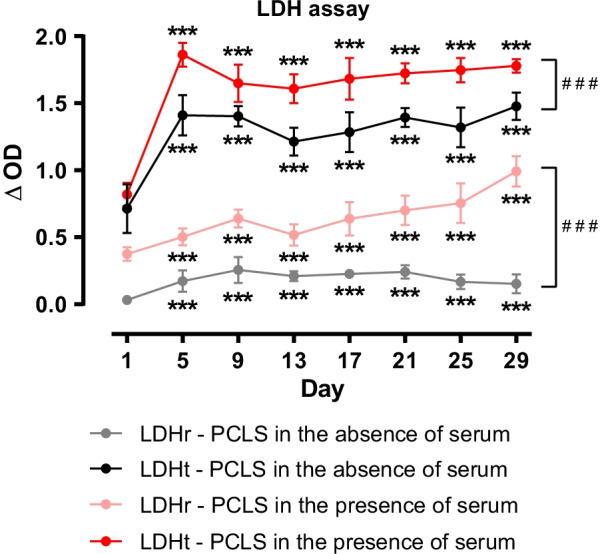
Measurement of lactate dehydrogenase (LDH) activity of long-term cultivated rat precision-cut lung slices (PCLS). Viability of tissue PCLS incubated in a standard culture medium (named PCLS in the absence of serum) and others incubated in a culture medium with the addition of 10% fetal bovine serum (FBS) (named PCLS in the presence of serum) was measured using LDH assay (optical density (OD) 492 nm, reference wavelength 595 nm). LDH release (LDHr) over 24 h as well as maximal LDH activity (= total LDH, LDHt) were analysed. Media without slices were used as negative controls, here no LDH activity could be observed. For each timepoint, measurements were performed in duplicates, n = 3. Data is expressed as mean ± standard deviation (SD). Statistics were carried out using the general mixed model analysis (PROC GLIMMIX, SAS 9.4, SAS Institute Inc., Cary, NC, USA), statistical differences to day 1 are indicated by asterisks (*** p < 0.001), statistical differences between the two groups (PCLS in the absence vs. PCLS in the presence of serum) are indicated by hashes (^###^p < 0.001)

Similar to WST-1 assay, LDH release (LDHr) of PCLS in the absence of serum was lowest on day 1 and remained on a significantly higher level thereafter, whereas PCLS in the presence of serum showed a slightly higher and increasing LDHr over time. Maximal LDH activity (LDHt) of PCLS in the absence of serum was lowest on day 1 after preparation and remained on a higher level for the rest of cultivation. Similar results were observed for PCLS in the presence of serum on a slightly higher level (Fig. 2). Relative cytotoxicity (measured by the ratio of LDHr/LDHt) of PCLS in the absence of serum remained below 20% (equals viability > 80%) over the whole cultivation period, PCLS in the presence of serum, however, showed markedly higher values (27.0–55.7%, equals 44.3–73.0% viability), especially from day 13 onwards (Additional file 3: Table S3).

### Histopathologic alteration

**Fig. 3:**
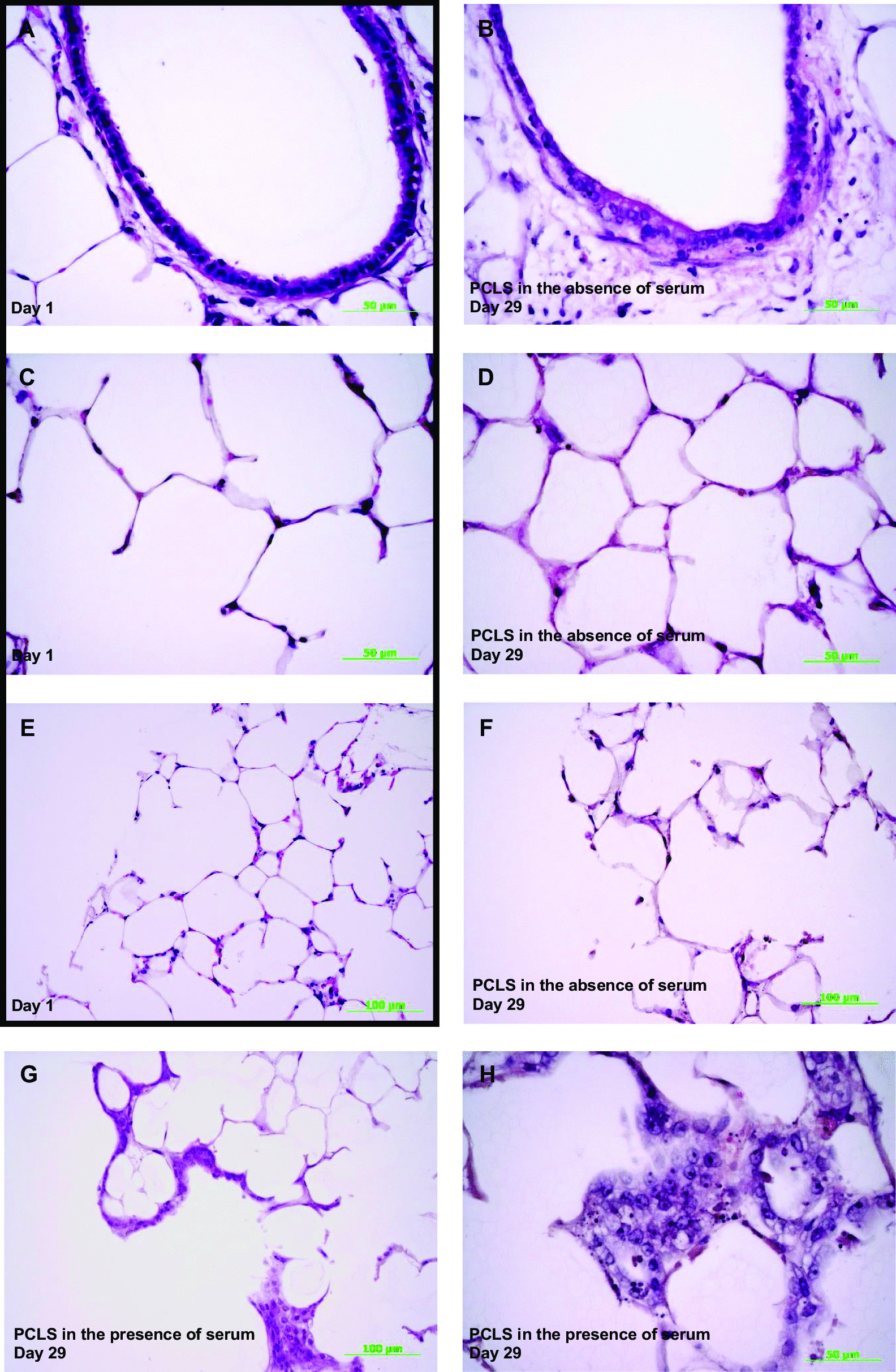
Exemplary illustration of hematoxylin–eosin (HE) stained long-term cultivated rat precision-cut lung slices (PCLS) on day 1 and 29. High-power magnification of the respiratory epithelium partly with cilia on day 1 (**A**) and day 29 (**B**) without any alterations. (**C**) shows normal alveolar epithelium on day 1 and (**D**) normal alveolar epithelium with some rare cell loss within the alveolar septa at day 29. While lung tissue in the cutting area shows no abnormalities on day 1 (**E**) and on day 29 incubated in standard culture medium (**F**), marked metaplastic/regenerative changes appear after 29 days of incubation with 10% fetal bovine serum (FBS) (**G**). The High-power magnification of **G** indicates metaplastic/regenerative cell groups with prominent nucleoli (**H**). Scale bars: 50 µm (**A**, **B**, **C**, **D**, **H**), 100 µm (**E**, **F**, **G**). Magnifications: 400x (**A**, **B**, C, **D**, **H**), 200x (**E**, **F**, **G**)

Both PCLS on day 1 (Fig. 3A) and day 29 (Fig. 3B) appeared normal regarding the respiratory epithelium and could be scored 0. Detachment of the respiratory epithelium occurred only in one of twelve slices in one airway after 29 days of incubation (score 1). The alveolar epithelium also showed no cell loss on day 1 (Fig. 3C, score 0), whereas on day 29 (Fig. 3D) the alveolar septa rarely showed cell loss (score 1). Furthermore, it was noticed that detachment of the endothelium of the blood vessels occurred in most of the sections after 29 days of incubation. Another focus of the evaluation was the lung tissue in the punch area. The tissue edges on day 1 showed no abnormal cells (Fig. 3E, score 0). Similarly, in five of six slices on day 29 after incubation with the standard culture medium, the lung tissue in the punch area appeared normal (Fig. 3F, score 0). Only one slice had histopathologically abnormal cells in a small area (score 1). In contrast, on day 29 after incubation with 10% FBS, these abnormal cells were found in at least half of the edge areas in all six preparations (Fig. 3G, score 2). At higher magnification (Fig. 3H), cell clusters with prominent nucleoli in the nucleus can be seen, which are presumably of metaplastic or regenerative origin. Airways located in the peripheral region also showed this alteration in the respiratory epithelium.

### Ability for bronchoconstriction

For analysing the reactivity of long-term cultivated rat PCLS, bronchoconstriction induced by ET-1, Ach and U46619 was measured.

**Fig. 4:**
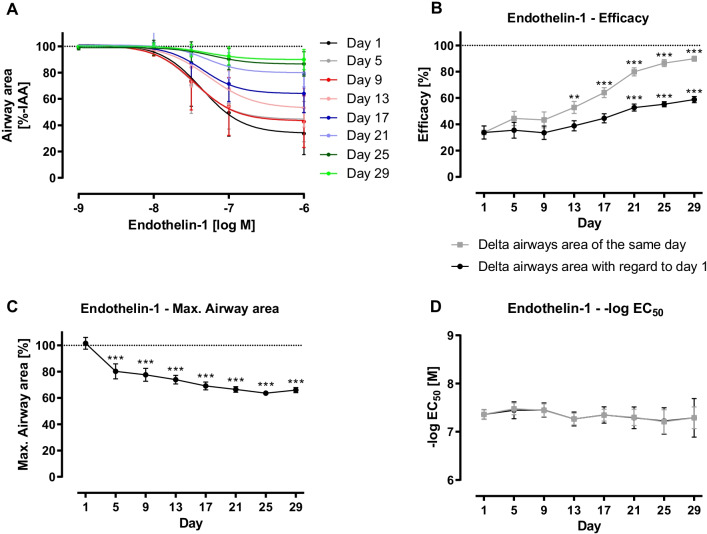
Endothelin-1 (ET-1)-induced bronchoconstriction in long-term cultivated rat precision-cut lung slices (PCLS). PCLS with intact airways and surrounding epithelium were used in measurements of ET-1-induced bronchoconstriction on measuring days. Different concentrations of ET-1 (10^–9^–10^–6^ M) were added to the slice, constriction was measured via microscope. Images were analysed with ImageJ. For each timepoint, measurements were performed in duplicates, n = 4, the same slices were used for measurements with ET-1 on every measuring day. Data is expressed as mean ± standard deviation (SD). First image of non-constricted airway of each measuring day was set as 100%. ET-1-induced reduction of airway area was expressed as percentage of the initial airway area (%-IAA) (**A**). Using GraphPad Prism 9, nonlinear regression with a four-parameter logistic equation was conducted from data of ET-1-induced bronchoconstriction (10^–9^–10^–6^ M). Maximal constriction (= efficacy) (**B**), maximal airway area (**C**) and half-maximal response (EC_50_) (**D**) were calculated. Analysis was performed based on control image of every measuring day (  ) and that of the first measuring day (●). Statistics were carried out using the general mixed model analysis (PROC GLIMMIX, SAS 9.4, SAS Institute Inc., Cary, NC, USA), statistical differences to day 1 are indicated by asterisks (**0.001 ≤ p < 0.01, ***p < 0.001)

The ability for bronchoconstriction after stimulation with increasing concentrations of ET-1 sustained over a period of 13 day long-term cultivation (Fig. 4A). However, a reduction in reactivity was seen (Fig. 4A + B). On day 1 in culture, airways showed a maximal constriction of 66.19% (equals 33.81% IAA) after stimulation with the highest concentration of ET-1, whereas it clearly decreased during long-term cultivation: On day 9, airways contracted by 56.67% (equals 43.33% IAA), on day 21 by 20.07% (equals 79.93% IAA) and on day 29 the contraction even reduced by half to 10.14% (equals 89.86% IAA) (Fig. 4A + B).

As shown in Fig. 4C, the IAA of day 1 could not be reached on any of the following measuring days but maximal airway area comprised 63.65% to 80.22% IAA. Besides, the sensitivity of the airways slightly decreased, as shown by increased EC_50_ values. On day 1 after preparation a dose of 43.68 nM ET-1 was sufficient to induce a 50% reduction of IAA, whereas a 17% higher dose (51.12 nM) was necessary to achieve the same IAA reduction on day 29 (Fig. 4D).

**Fig. 5:**
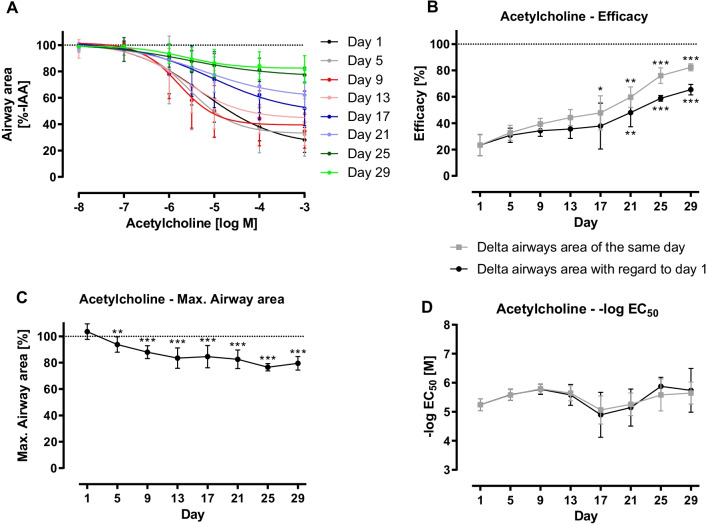
Acetylcholine (Ach)-induced bronchoconstriction in long-term cultivated rat precision-cut lung slices (PCLS). PCLS with intact airways and surrounding epithelium were used in measurements of Ach-induced bronchoconstriction on measuring days. Different concentrations of Ach (10^–8^–10^–3^ M) were added to the slice, constriction was measured via microscope. Images were analysed with ImageJ. For each timepoint, measurements were performed in duplicates, n = 4, the same slices were used for measurements with Ach on every measuring day. Data is expressed as mean ± standard deviation (SD). First image of non-constricted airway of each measuring day was set as 100%. Ach-induced reduction of airway area was expressed as percentage of the initial airway area (%-IAA) (**A**). Using GraphPad Prism 9, nonlinear regression with a four-parameter logistic equation was conducted from data of Ach-induced bronchoconstriction (10^–8^–10^–3^ M). Maximal constriction (= efficacy) (**B**), maximal airway area (**C**) and half-maximal response (EC_50_) (**D**) were calculated. Analysis was performed based on control image of every measuring day (  ) and that of the first measuring day (●). Statistics were carried out using the general mixed model analysis (PROC GLIMMIX, SAS 9.4, SAS Institute Inc., Cary, NC, USA), statistical differences to day 1 are indicated by asterisks (*0.01 ≤ p < 0.05; **0.001 ≤ p < 0.01, ***p < 0.001)

Similar to the results of ET-1-induced bronchoconstriction, CRC of Ach showed a sustained ability for bronchoconstriction for 17 days, a reduction in reactivity, a decrease in maximal airway area and a slight alteration regarding sensitivity (Fig. 5).

**Fig. 6:**
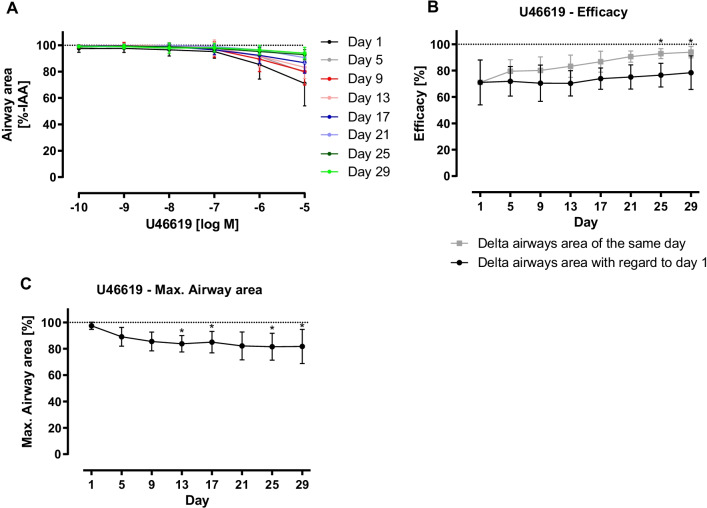
9,11-dideoxy-9α,11α-methanoepoxy prostaglandin F_2α_ (U46619)-induced bronchoconstriction in long-term cultivated rat precision-cut lung slices (PCLS). PCLS with intact airways and surrounding epithelium were used in measurements of U46619-induced bronchoconstriction on measuring days. Different concentrations of U46619 (10^–10^–10^–5^ M) were added to the slice, constriction was measured via microscope. Images were analysed with ImageJ. For each timepoint, measurements were performed in duplicates, n = 4, the same slices were used for measurements with U46619 on every measuring day. Data is expressed as mean ± standard deviation (SD). First image of non-constricted airway of each measuring day was set as 100%. U46619-induced reduction of airway area was expressed as percentage of the initial airway area (%-IAA) (**A**). Using GraphPad Prism 9, nonlinear regression with a four-parameter logistic equation was conducted from data of U46619-induced bronchoconstriction (10^–10^–10^–5^ M). Maximal constriction (= efficacy) (**B**) and maximal airway area (**C**) were calculated. Analysis was performed based on control image of every measuring day (  ) and that of the first measuring day (●). Due to non-sigmoid characteristic, calculation of half-maximal response (EC_50_) was not possible. Statistics were carried out using the general mixed model analysis (PROC GLIMMIX, SAS 9.4, SAS Institute Inc., Cary, NC, USA), statistical differences to day 1 are indicated by asterisks (*0.01 ≤ p < 0.05)

In contrast to ET-1 and Ach, PCLS only showed a weak response after stimulation with increasing concentrations of U46619. Concentrations of 10^–10^ to 10^–7^ M caused almost no bronchoconstriction at all, while airways contracted at higher concentrations. Reactivity markedly decreased during long-term cultivation (Fig. 6).

### Gene expression

Based on the mediators for CRCs, specific target genes (Additional file 1: Table S2) were chosen and alterations in gene expression during long-term cultivation of PCLS were investigated by RT-qPCR. Furthermore, possible effects of PCLS’ preparation on gene expression were analysed by comparing mRNA expression of unfilled lung tissue (*Lobus accessorius*) with PCLS from the preparation day.

#### ***Changes in gene expression pattern during long***-***term cultivation***

**Fig. 7:**
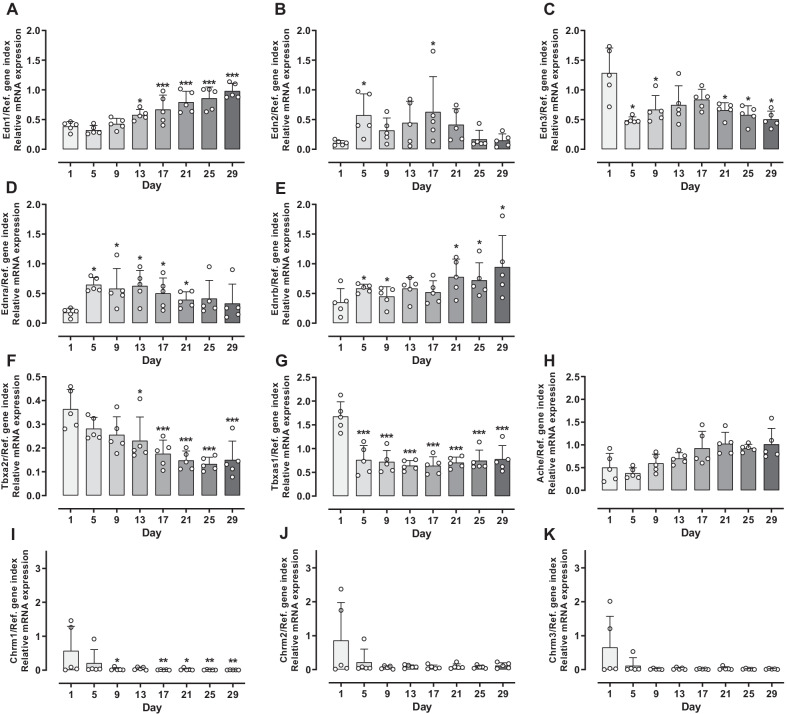
Gene expression of long-term cultivated rat precision-cut lung slices (PCLS). PCLS were analysed for messenger ribonucleic acid (mRNA) expression of endothelin-1 (Edn1, **A**), endothelin-2 (Edn2, **B**), endothelin-3 (Edn3, **C**), endothelin receptor type A (Ednra, **D**), endothelin receptor type B (Ednrb, **E**), thromboxane A_2_ receptor (Tbxa2r, **F**), thromboxane A synthase 1 (Tbxas1, **G**), acetylcholinesterase (Ache, **H**), muscarinic acetylcholine receptor M1 (Chrm1, **I**), muscarinic acetylcholine receptor M2 (Chrm2, **J**) and muscarinic acetylcholine receptor M3 (Chrm3, **K**) in relation to a reference gene index (Ref. gene index) consisting of ribosomal protein lateral stalk subunit P0 (Rplp0) and beta-actin (Actb). For this purpose, PCLS were snap frozen in liquid nitrogen after denoted cultivation time, ribonucleic acid (RNA) isolation was performed by a combination of phenol–chloroform extraction and magnetic-bead technique. Afterwards, reverse transcription (RT) and quantitative polymerase chain reaction (qPCR) were performed. For each timepoint, measurements were performed in triplets, n = 5, per timepoint an average of 6–8 slices was snap frozen in liquid nitrogen, of which 100 mg of the slices were used for RNA isolation. RT-qPCR was performed with an input of 230 ng RNA. Data is expressed as mean + standard deviation (SD), dots represent individual data points. Statistics were carried out using the general mixed model analysis (PROC GLIMMIX, SAS 9.4, SAS Institute Inc., Cary, NC, USA), statistical differences to the control group (PCLS of day 1) are indicated by asterisks (*0.01 ≤ p < 0.05; **0.001 ≤ p < 0.01, ***p < 0.001)

Regarding endothelin-1 (Edn1), mRNA expression of day 1 to day 9 maintained a constant level, but expression significantly increased thereafter (Fig. 7A). With regard to endothelin-2 (Edn2), which was barely detectable on day 1, an upregulation in expression was observed between days 5 and 21 followed by a decrease to the base level (Fig. 7B). In comparison to the control group (PCLS of day 1) endothelin-3 (Edn3) was expressed on a significantly lower level on days 5 and 9 (reduction of > 50%), increased slightly until day 17 to a level that was still lower than that of day 1 to decrease again until the end of cultivation time (Fig. 7C). Compared to the control group, mRNA expression of endothelin receptor type A (Ednra), was upregulated for the whole period of long-term incubation with a significantly higher expression on days 5 to 21 (Fig. 7D). Regarding endothelin receptor type B (Ednrb), mRNA expression was significantly upregulated on day 5, stayed constant afterwards and increased slightly to the end of cultivation time (Fig. 7E). In comparison to the control group thromboxane A_2_ receptor (Tbxa2r) showed a lower and consistently declining expression over time in culture with significantly lower expression from day 13 onwards (Fig. 7F). Regarding thromboxane A synthase 1 (Tbxas1), expression was significantly downregulated on day 5 and maintained a significant lower but constant level for the rest of long-term cultivation (Fig. 7G). mRNA expression of acetylcholinesterase (Ache) was similar in its expression pattern to that of Edn1 with a constant level of gene expression until day 9 and a slight upregulation thereafter (Fig. 7H). Regarding the muscarinic acetylcholine receptors M1-3 (Chrm1, Chrm2, Chrm3) very low gene expression levels were observed with a few outliers on days 1 and 5 (Fig. 7I–K). All three genes, including Chrm3, the primary receptor for bronchoconstriction after stimulation with Ach [30], were hard to detect, possibly because of low basal expression. Despite an additional DNase digestion, the no reverse transcriptase (NRT) controls were positive with a Ct > 30. With normal gene expression of the target gene, this should not influence the result. Since the expression of the muscarinic receptors was low and in some samples also in the range of a Ct of 30, the results of the gene expression should be taken with caution.

#### Effects of PCLS’ preparation on gene expression pattern

Apart from alterations in gene expression levels during long-term cultivation of PCLS, it was investigated whether the preparation of PCLS (agarose filling, slicing, media changes for 4 h) itself might effect gene expression.

**Fig. 8:**
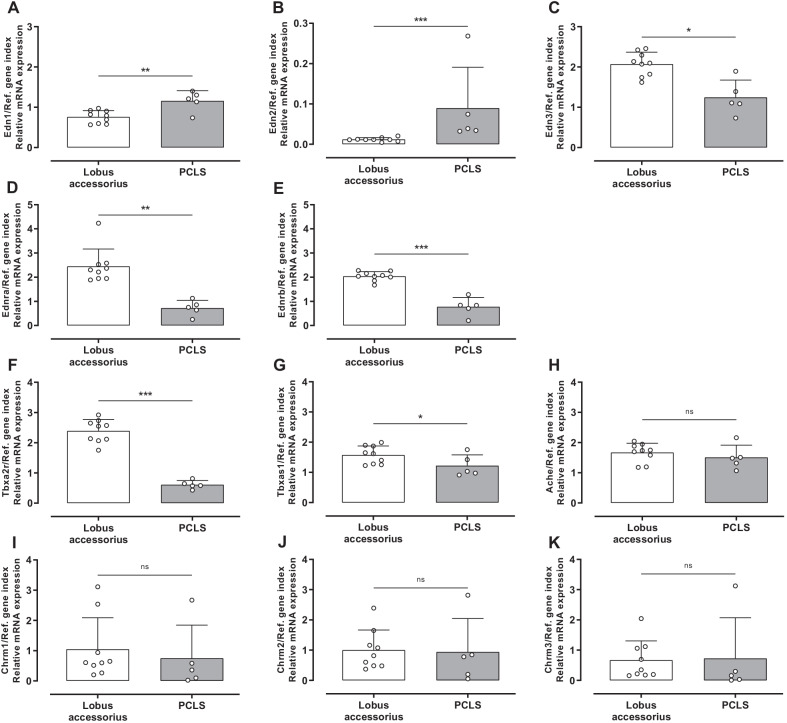
Gene expression of rat native lung tissue compared to rat precision-cut lung slices (PCLS). Native lung tissue (*Lobus accessorius*) and PCLS of the preparation day were analysed for messenger ribonucleic acid (mRNA) expression of endothelin-1 (Edn1, **A**), endothelin-2 (Edn2, **B**), endothelin-3 (Edn3, **C**), endothelin receptor type A (Ednra, **D**), endothelin receptor type B (Ednrb, **E**), thromboxane A_2_ receptor (Tbxa2r, **F**), thromboxane A synthase 1 (Tbxas1, **G**), acetylcholinesterase (Ache, **H**), muscarinic acetylcholine receptor M1 (Chrm1, **I**), muscarinic acetylcholine receptor M2 (Chrm2, **J**) and muscarinic acetylcholine receptor M3 (Chrm3, **K**) in relation to a reference gene index (Ref. gene index) consisting of ribosomal protein lateral stalk subunit P0 (Rplp0) and beta-actin (Actb). For this purpose, native lung tissue and PCLS of the preparation day were snap frozen in liquid nitrogen after withdrawal and PCLS preparation, respectively. Ribonucleic acid (RNA) isolation was performed by a combination of phenol–chloroform extraction and magnetic-bead technique. Afterwards, reverse transcription (RT) and quantitative polymerase chain reaction (qPCR) were performed. Measurements were performed in triplets, an average of 6–8 slices (n = 5) and native lung tissue of *Lobus accessorius* (n = 9) were snap frozen in liquid nitrogen, of which 100 mg of the slices and 20 mg of the lobes, respectively, were used for RNA isolation. RT-qPCR was performed with an input of 230 ng RNA. Data is expressed as mean + standard deviation (SD), dots represent individual data points. Statistics were carried out using the general mixed model analysis (PROC GLIMMIX, SAS 9.4, SAS Institute Inc., Cary, NC, USA), statistical differences to the control group (*Lobus accessorius*) are indicated by asterisks (*0.01 ≤ p < 0.05; **0.001 ≤ p < 0.01, ***p < 0.001, *ns* no significance)

As shown in Fig. 8, significant differences in gene expression were observed regarding Edn1, Edn2, Edn3, Ednra, Ednrb, Tbxa2r and Tbxas1: mRNA expression of Edn1 and Edn2 has been upregulated after preparation, whereas Edn3, Ednra and Ednrb, Tbxa2r and Tbxas1 were expressed on significantly lower levels in PCLS than in native lung tissue. In contrast, the process of preparation seemed to not influence gene expression of Ache, Chrm1, Chrm2 and Chrm3. However, as positive NRTs the results of Chrm1-3 had to be handled with care.

## Discussion

In most studies PCLS experiments were conducted under short-term culturing conditions (≤ 72 h) [7, 13, 23]. Therefore, results were limited to acute effects of drugs or chemicals. Apart from these short-term experiments there is a large interest in research of chronic effects of drugs as well as investigation of mechanisms and methods of treatment regarding chronic airway diseases. Thus, we investigated long-term cultivated rat PCLS regarding viability, histopathology, reactivity and gene expression to establish a model useful for in-depth study of chronic diseases like pulmonary hypertension, fibrosis or chronic inflammation and chronic pulmonary toxicity in the future.

While another research group used long-term cultivation of human PCLS over a period of 2 weeks [24, 25] and just recently over 4 weeks [26], we successfully cultivated rat PCLS over 29 days.

### Viability of long-term cultivated PCLS

In terms of metabolic activity PCLS cultivated in standard culture medium (without serum) showed no significant decrease in WST-1 assay over 29 days, as shown by others for up to 28 days [25, 26, 31]. In accordance with Temann et al. 2017 [25] PCLS on day 1 after preparation had the lowest metabolic activity, due to a recovery phase after the slicing procedure.

As LDH assays of long-term cultivated slices show, LDHr as well as LDHt of PCLS without serum increased from day 1 to day 5 and stayed stable afterwards. The rise of LDHt could indicate an increase in total cells during the first five days in culture or a reorganisation of the cells after the slicing process, as this preparation leads to injured cells which may reseal. Stable values of LDHt from day 5 onwards, may indicate a constant amount of cells thereafter. Nevertheless, one cannot exclude that a (de)differentiation of cell types occurs, as specific cell type proliferates while others decrease. This is supported by others, where decrease in cytokine production during 2-week cultivation [25] and loss of pneumocytes and endothelial cells during 4-week cultivation of human PCLS [26] was found.

We showed that relative cytotoxicity over long-term cultivation of 29 days amounted to less than 20% (equals viability > 80%), which was reported by others for shorter incubation times [5, 25, 26].

### Cultivation in presence of serum

To determine viability of PCLS in the presence of 10% FBS (PCLS with serum), WST-1 and LDH tests were performed. There, stronger metabolic activity (WST-1) was observed after day 9. Song et al. 2020 [32] reported circulating mitochondria in FBS, even after heat inactivation. However, this should affect the WST-1 test at all days of the incubation time. Alternatively, proliferation or differentiation of cell types with higher mitochondrial activity after day 9 could be accounted for, as already shown for fibroblasts [33]. PCLS in the presence of serum displayed higher LDH levels also compared to the serum-free controls. As LDHt from day 5 onwards did not change, an increase in relative cytotoxicity (measured by the ratio of LDHr/LDHt) indicates that cultivation of PCLS with 10% FBS might have led to a higher number of damaged cells that release LDH into the culture medium. Higher numbers of damaged cells indicate a higher LDHr. However, LDHt did not decline accordingly, which could be explained by cell proliferation. Heat inactivation should inactivate complement factors and thus prevent complement-mediated cell lysis [34]. However, FBS contains only a small amount of complement factors [35] which is even reduced further through heat inactivation.

Unlike Henjakovic et al. 2008 [23], who reported an improvement of murine PCLS’ viability by the addition of FBS (1%, 3%, 10%) for longer than 24 h, we could not observe positive effects regarding viability of long-term cultivated PCLS.

### Histopathologic alteration

Consistent with the results of Preuß et al. 2022 [26], the PCLS sections showed no changes in the respiratory epithelium and minimal alterations in the alveolar epithelium after 29 days of incubation. In contrast to their results [26], in serum-free incubation medium only isolated rare histopathological changes in the punch area were observed, whereas in the presence of serum distinct cellular changes of metaplastic and/or regenerative origin without keratinization could be found. It should clearly be underlined that these metaplastic changes are not to be interpreted as precursors of malignant processes. In addition, a specialized stain for connective tissue showed no significant increase in collagen fibers in the altered tissue areas.

### Reactivity of long-term cultivated PCLS

Based on the results of CRCs, PCLS remained partly functional during the period of long-term cultivation. However, reactivity decreased (Ach, ET-1, U46619), as seen by the altered maximal bronchoconstriction values and altered (Ach) or rather declined (ET-1, U46619) sensitivity. The difference by the mediators may be due to a rapid reversible (Ach) or sustained contraction (ET-1, U46619), even after the washing process. Even though the EC_50_ values for Ach and ET-1 are not significantly different, we find reproducible contractility until day 13 (ET-1), day 17 (Ach) and day 25 (U46619) as afterwards there are significant differences in the maximal airway contraction compared to day 1.

Neuhaus et al. 2017 [24] reported a sustained ability of methacholine (Mch)-induced bronchoconstriction, decreased reactivity and sensitivity over a 15-day cultivation of human PCLS. Likewise, Li et al. 2020 [36] showed preserved ASM contraction of murine PCLS after Mch- and serotonin-stimulation over a period of 2 weeks in the presence of insulin, a pleiotropic growth factor hormone [37].

The contraction by ET-1 showed similar EC_50_ values (43.68 nM) over the whole incubation time, even though the maximal contraction was reduced with incubation time. The EC_50_ values for large airways are in the same range as in our former publications (58 nM, 63 nM and 22 nM) [6, 38]. The thromboxane receptor agonist U46619 induced a weak bronchoconstriction to 70% IAA. In contrast, a significant bronchoconstriction started at 1 µM.

Overall, there is a reduced contractility for all mediators (Ach, ET-1, U46619) with increasing incubation time. This may be to due to a decreased relaxation of airways. Several reasons for this are conceivable: First, airways are embedded in parenchyma which exhibits tethering forces on the airways increasing with contraction [27]. In PCLS the alveolar space is stabilised by undissolved agarose, which slowly dissolves as indicated by shrinking of the PCLS (data not shown). This indicates that tethering forces decrease. Here, additional stretch of the tissue may be beneficial to maintain the maximal relaxation and maybe increase viability. Second, there could be less dilation of the airways after the first application of the mediators due to sustained (pre-)contraction. Third, an increased receptor expression as found for the Edn1 gene could be a cause. Last, ET-1 induces the formation/release of thromboxane, which leads to a sustained contraction of airways. Even the thromboxane production on mRNA level did not change over time, the stability of the mRNA and by this the amount of protein could be affected.

### RNA isolation of PCLS

Preliminary tests have shown that RNA from PCLS could neither be successfully isolated using a column-based isolation procedure nor using conventional phenol–chloroform extraction. Niehof et al. 2017 [39] optimised RNA isolation from PCLS. We further optimised the protocol by the following alterations: 1. No use lysis buffer. 2. An extraction without isoamyl alcohol. 3. Use of 1.5 ml tubes for the magnetic beads. 4. Additional DNase treatment step. Compared to rat PCLS of Niehof et al. 2017 [39] our RNA yield was more than twice as high (77.60 ± 27.71 ng/µl vs. 35.1 ± 21.2 ng/µl) and purity comparable (A_260_/A_280_ ratio 2.039 ± 0.073 vs. 1.96 ± 0.09), respectively.

### Gene expression

Only few papers have been published on gene expression analysis of short-term cultured PCLS [28, 39–41]. To our knowledge, no publication showed gene expression studies of long-term cultured PCLS.

Besides ET-1, endothelin-2 (ET-2) and endothelin-3 (ET-3) induce a weak bronchoconstriction [42]. ET-2 seems to be expressed markedly lower than the other endothelins and thus can induce the mentioned effects to a slighter extent. Endothelin receptors type A (ET_A_) and B (ET_B_) are widely spread in pulmonary tissue [43], inducing vasoconstriction or bronchoconstriction, respectively. Both effects can be attenuated by the other receptor, e.g. ET_B_-induced bronchoconstriction is attenuated by bronchodilatory mechanisms of ET_A_ [44]. As shown in the results of qPCR, the expression of ET_B_ slightly increased over time, whereas that of ET_A_ declined, which should result in increased bronchoconstriction. However, CRCs of ET-1 show a contrary result. One explanation would be a desensitization by increased precontraction due to increased endogenous production of endothelins, as shown by the increased ET-1 mRNA expression.

The significant decrease in expression of Tbxa2r is in line with the results of U46619, leading to a declined bronchoconstriction over cultivation time. Tbxas1 expression was significantly downregulated on day 5 and maintained a lower, constant level. Besides blood platelets, Tbxa2r and Tbxas1 are expressed in cells of the mononuclear phagocyte system (e.g. macrophages) and endothelial cells [45, 46] and their reduction due to the daily change of media would be conceivable. However, Neuhaus et al. 2017 [24] still reported a high number of intact macrophages within tissue over 15-day cultivation.

CRCs of Ach showed a decrease in reactivity as well as reduced relaxation of PCLS, similar to those of ET-1 and U46619. This could be due to either regulation of M2 and M3 receptor or an increased breakdown of Ach by Ache. However, after day 1 the expression of muscarinic receptors was very low, without changes over incubation time. The main problem regarding the muscarinic receptors were the positive negative controls during qPCR which may indicate genomic contamination, even after an additional DNase step. For the genes of the muscarinic receptors no primers could be designed on the intron–exon boundaries. Therefore, the results must be handled with care.

However, an increased breakdown of Ach by Ache might be supported by an increase of Ache gene expression during cultivation.

Of note, the amount of cells during long-term cultivation is kept in balance, a differentiation of cell types could occur with specific cell types proliferating and others dying as reported by Preuß et al. 2022 [26]. This might also influence gene expression pattern of the investigated genes; it could even influence the function of cells.

In addition, the effects of PCLS preparation (filling and slicing) on gene expression is poorly analysed. The filling of lungs with large volumes of agarose could induce volutrauma or atelectrauma [47]. However, this process involves infiltration of immune cells, which is not present in the PCLS [48]. As shown by qPCR, the process of slicing seems to influence mRNA expression of most target genes. As several PCLS were available from the same lungs, changes of the PCLS due to the production process are balanced by internal controls (untreated slices).

PCLS stay viable and functional as confirmed here and by other groups [5, 24, 25].

To investigate an induction and release of cytokines or growth factors due to preparation, tests with native and agarose-containing tissue as well as PCLS from different stages of preparation (after slicing and media changes, respectively) need to be performed.

## Conclusion

In summary, PCLS were successfully cultivated over a 29-day time period with sustained viability. The ability of bronchoconstriction was maintained between 13 and 25 days, depending on the mediator. Increasing respiratory tone presumably ascribes to decreasing stabilisation of the lung tissue over time. Regarding transcription, alteration in gene expression of the investigated target genes was observed during long-term cultivation with mixed results. Furthermore, the preparation of PCLS seems to influence gene expression of most target genes. The addition of FBS to the culture medium did not improve viability of PCLS, even though FBS seems to result in marked cellular changes of metaplastic and/or regenerative origin.

We showed that 29-day long-term rat PCLS cultivation is feasible. Thus, it should be possible to use PCLS for in-depth study of chronic diseases like pulmonary hypertension, fibrosis or chronic inflammation as well as chronic pulmonary toxicity.

## Supplementary Information

**Additional file 1: Table S1**. Concentration of components within the culture medium.

**Additional file 2: Table S2**. Used primers (F: forward primer, R: reverse primer) and their annealing temperature for RT-qPCR.

**Additional file 3: Table S3**. Measurement of lactate dehydrogenase (LDH) activity of long-term cultivated rat precision-cut lung slices (PCLS). Viability of tissue PCLS incubated in a standard culture medium (named PCLS in the absence of serum) and others incubated in a culture medium with the addition of 10% fetal bovine serum (FBS) (named PCLS in the presence of serum) was measured using LDH assay (optical density (OD) 492 nm, reference wavelength 595 nm). For each timepoint, measurements were performed in duplicates, n = 3. For calculation of relative cytotoxicity, the ratio of LDH release (LDHr) over 24 h/maximal LDH activity (= total LDH, LDHt) was determined. Data is expressed as mean ± standard deviation (SD).

## Data Availability

The datasets generated and/or analysed during the current study are available from the corresponding author on reasonable request.

## Abbreviations

Ach: Acetylcholine
Ache: Acetylcholinesterase
Actb: Beta-actin
ASM: Airway smooth muscle
CaCl_2_ × 2 H_2_O: Calcium chloride dihydrate
Chrm1: Muscarinic acetylcholine receptor M1
Chrm2: Muscarinic acetylcholine receptor M2
Chrm3: Muscarinic acetylcholine receptor M3
COPD: Chronic obstructive pulmonary disease
CRC: Concentration–response-curve
DNase: Deoxyribonuclease
EC_50_: Half-maximal response
Ednra: Endothelin receptor type A
Ednrb: Endothelin receptor type B
Edn1: Endothelin-1
Edn2: Endothelin-2
Edn3: Endothelin-3
ET_A_: Endothelin receptor type A
ET_B_: Endothelin receptor type B
ET-1: Endothelin-1
ET-2: Endothelin-2
ET-3: Endothelin-3
FBS: Fetal bovine serum
HE: Hematoxylin and eosin
HEPES: N-(2-Hydroxyethyl)piperazine-N′-(2-ethanesulfonic acid), 4-(2-Hydroxyethyl)piperazine-1-ethanesulfonic acid
IAA: Initial airway area
KCl: Potassium chloride
LDH: Lactate dehydrogenase
LDHr: LDH release
LDHt: Total LDH
Mch: Methacholine
MEM: Minimal essential medium
MgSO_4_ × 7 H_2_O: Magnesium sulfate heptahydrate
mRNA: Messenger ribonucleic acid
NaCl: Sodium chloride
NaHCO_3_: Sodium bicarbonate
NaH_2_PO_4_ x H_2_O: Disodium hydrogen phosphate solution
NRT: No reverse transcriptase control
OD: Optical density
PCLS: Precision-cut lung slices
Ref. gene index: Reference gene index
RNA: Ribonucleic acid
Rplp0: Ribosomal protein lateral stalk subunit P0
RT: Reverse transcription
qPCR: Quantitative polymerase chain reaction
SD: Standard deviation
Tbxas1: Thromboxane A synthase 1
Tbxa2r: Thromboxane A_2_ receptor
U46619: 9, 11-Dideoxy-9α, 11α-methanoepoxy prostaglandin F_2α_
WST: Water soluble tetrazolium
