# Supplementary material for: Functional changes in long-term incubated rat precision-cut lung slices
Source: Respir Res. 2022 Sep 20;23:261. doi: 10.1186/s12931-022-02169-5 (PMC9490993; doi:10.1186/s12931-022-02169-5)
Supplement: Supplementary file 1 — Additional file 1: Table S1. Concentration of components within the culture medium. [file 12931_2022_2169_MOESM1_ESM.pdf]

**Eagle's Balanced Salt Solution**		
$CaCl_2$ x 2 H_2O	Sigma-Aldrich Chemie GmbH, Steinheim, Germany	0.265 g/l
$MgSO_4$ x 7 H_2O	Sigma-Aldrich Chemie GmbH, Steinheim, Germany	0.2 g/l
KCl	Sigma-Aldrich Chemie GmbH, Steinheim, Germany	0.4 g/l
NaCl	Sigma-Aldrich Chemie GmbH, Steinheim, Germany	6.8 g/l
NaH_2PO_4 x H_2O	Sigma-Aldrich Chemie GmbH, Steinheim, Germany	0.14 g/l
**Other components**		
Glucose	Sigma-Aldrich Chemie GmbH, Steinheim, Germany	3.0 g/l
$NaHCO_3$	Sigma-Aldrich Chemie GmbH, Steinheim, Germany	2.2 g/l
HEPES	Carl Roth GmbH + Co. KG, Karlsruhe, Germany	6.0 g/l
**Solutions**		
Sodium pyruvate, 100 mM	Capricorn Scientific GmbH, Ebsdorfergrund, Germany	10 ml/l
L-glutamine, 200 mM	Capricorn Scientific GmbH, Ebsdorfergrund, Germany	10 ml/l
MEM amino acids (50x)	Sigma-Aldrich Chemie GmbH, Steinheim, Germany	20 ml/l
MEM vitamins (100x)	Sigma-Aldrich Chemie GmbH, Steinheim, Germany	10 ml/l
Penicillin (10.000 U/ml) and streptomycin (10 mg/ml)	Sigma-Aldrich Chemie GmbH, Steinheim, Germany	10 ml/l
