# Supplementary material for: Functional changes in long-term incubated rat precision-cut lung slices
Source: Respir Res. 2022 Sep 20;23:261. doi: 10.1186/s12931-022-02169-5 (PMC9490993; doi:10.1186/s12931-022-02169-5)
Supplement: Supplementary file 2 — Additional file 2: Table S2. Used primers (F: forward primer, R: reverse primer) and their annealing temperature for RT-qPCR. [file 12931_2022_2169_MOESM2_ESM.pdf]

Gene	Sequence	Annealing temperature
Ache	F: CTATGCCTACATCTTTGAACAC<br>R: CACCGTGGAGACTTAGAG	56 °C
Actb	F: CTTCCTTCCTGGGTATGGAATCCT<br>R: GGATAGAGCCACCACACAC	56 °C
Chrm1	F: TGTCAGTCCCAACATCAC<br>R: CTTGAAGGAGATGAGTACCA	59 °C
Chrm2	F: CAATGCCTCCGTTATGAATCTC<br>R: CCCTACGATGAACTGCCA	56 °C
Chrm3	F: GGTCATCTCCTTTGTCCT<br>R: CAGTTTCCTTATAGATCCTCCA	56 °C
Ednra	F: CGTGGTCATTGATCTCCC<br>R: CTGCTCTGTACCTGTCCA	57 °C
Endrb	F: GACCAGAGCAATCCTCAG<br>R: CACAAACACGACTTAAAGCAG	57 °C
Edn1	F: GAACTCCGAGCCCAAAGTACC<br>R: GAGGTCTTGATGCTGTTGCTG	57 °C
Edn2	F: TGGACATCATCTGGGTGAACACTG<br>R: CAGCAGAAGAGCACTCGCA	59 °C
Edn3	F: AGACTGTGCCCTATGGAC<br>R: CCTTGACTTCAGCCTTTGAC	56 °C
Rplp0	F: ACAGTACCTGCTCAGAACACC<br>R: TGCCATTGTCAAACACCTGCT	56 °C
Tbxas1	F: TTGAACGAGATGACACCT<br>R: AGTAACACCTCTGGATGTC	56 °C
Tbxa2r	F: CCTTGCTGGTCTTCATCCT<br>R: TGGGAAGTGAACCTTGGA	56 °C
