# Supplementary figures and images for: Functional changes in long-term incubated rat precision-cut lung slices
Source: Respir Res. 2022 Sep 20;23:261. doi: 10.1186/s12931-022-02169-5 (PMC9490993; doi:10.1186/s12931-022-02169-5)

## Ratio LDHr/LDHt

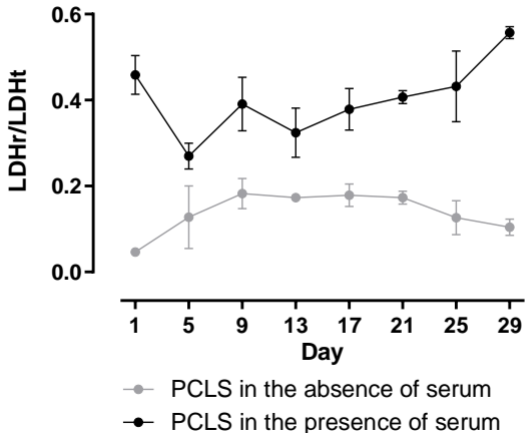

Supplement: Supplementary file 3 — Additional file 3: Table S3. Measurement of lactate dehydrogenase (LDH) activity of long-term cultivated rat precision-cut lung slices (PCLS). Viability of tissue PCLS incubated in a standard culture medium (named PCLS in the absence of serum) and others incubated in a culture medium with the addition of 10% fetal bovine serum (FBS) (named PCLS in the presence of serum) was measured using LDH assay (optical density (OD) 492 nm, reference wavelength 595 nm). For each timepoint, measurements were performed in duplicates, n = 3. For calculation of relative cytotoxicity, the ratio of LDH release (LDHr) over 24 h/maximal LDH activity (= total LDH, LDHt) was determined. Data is expressed as mean ± standard deviation (SD). [file 12931_2022_2169_MOESM3_ESM.pdf]
